# Differential effects of high-fat diet on salivary and gut microbiota

**DOI:** 10.3389/fcimb.2025.1547555

**Published:** 2025-02-24

**Authors:** Jingxuan Bai, Yixue Tian, Yujia Lu, Yuke Chen, Min Yu, Xuemei Gao

**Affiliations:** ^1^ Department of Orthodontics, Peking University School and Hospital of Stomatology, Beijing, China; ^2^ Center for Oral Therapy of Sleep Apnea, Peking University Hospital of Stomatology, Beijing, China

**Keywords:** obesity, 16S rRNA, oral microbiome, gut microbiome, oral-gut axis

## Abstract

**Objectives:**

Microorganisms contribute to the pathogenesis of obesity, while more studies focus on gut microbiome. However, the relationship between oral microbiota and obesity has yet to be elucidated. This study was designed to investigate the similarities and differences in the effects of a high-fat diet on salivary and gut microbiota through mouse experiments, exploring the hypothesis that oral microbial mechanisms may contribute to obesity.

**Methods:**

An obese mouse model was established in male C57BL/6J mice by feeding a high-fat diet, confirmed by body weight records and blood glucose tests. This study evaluated the physiological effects of the high-fat diet on mice. 16S rRNA sequencing technology was used to analyze changes in salivary and gut microbiota, and gas chromatography-mass spectrometry was employed to evaluate 17 short-chain and medium-chain fatty acids quantitatively.

**Results:**

The microbiota distribution in salivary was different between the high-fat diet (HFD) and normal chow diet (NCD) groups. At the genus level of salivary microbiota, *Streptococcus* and *Escherichia* were highly abundant in the HFD group. *Rodentibacter* and *Turicibacter* were more abundant in the NCD group. Regarding the gut microbiome, the diversity changes of gut microbiota are more significant than those of salivary microbiota. The HFD group had a significantly higher abundance of *Kineothrix*, *Cryptobacteroides*, and a lower abundance of *CAG-485*. Nine genera had consistent alterations in salivary and gut microbiota, among which *Akkermansia*, *Lactobacillus*, and *Intestinimonas* were significantly correlated with physiological indicators, and *Muribaculum* was significantly correlated with increased decanoic acid levels in the HFD group. The dysregulated nine genera were associated with significant upregulation of certain metabolic pathways of the HFD group, including the pentose phosphate, bacterial invasion of epithelial cells, and steroid biosynthesis pathways.

**Conclusions:**

There are differences and similarities in the effects of HFD on salivary and gut microbiota. Certain genera of the oral-gut axis altered consistently by HFD may affect obesity through mechanisms involving metabolic pathways and inflammation.

## Introduction

Obesity is an emerging epidemic globally, garnering widespread attention due to its co-occurrence with comorbidities, including diabetes, hypertension, and coronary heart disease. Diet was found to be a risk factor for obesity (Van Der Merwe et al., 2020).

Current evidence suggests diet may cause obesity by altering the gut microbiome. Animal experiments indicated that the high-fat diet, not the obese state, mainly accounted for the large alterations in the microbiome composition (Hildebrandt et al., 2009), consistent with other studies, which also observed that the compositional changes in the fecal microbiota were primarily a feature of high-fat feeding rather than genetically induced obesity (Murphy et al., 2010). Emerging evidence suggests that the gut microbiome plays a vital role in obesity (Liu et al., 2017a). Germ-free mice receiving fecal transplants from obese human donors showed increased fat deposition and metabolic complications, suggesting an association between gut microbiome composition and the pathophysiology of obesity (Ridaura et al., 2013). Besides, cohousing mice harboring an obese twin’s microbiota (Ob) with mice containing the lean co-twins’ microbiota prevented the development of increased body mass in Ob cage mates due to changes in microbiome and Short-chain fatty acids (SCFAs). SCFAs, as beneficial microbial metabolites for preventing and treating glucose and lipid metabolism disorders, participate in maintaining intestinal mucosal integrity, improving glucose and lipid metabolism, controlling energy consumption, regulating fat storage, immune system, and inflammatory response (Murphy et al., 2010; Canfora et al., 2019; Agus et al., 2021). The causal relationship between gut microbiome and obesity is unidirectional. A high-fat diet alters the gut microbiome, which further induces obesity through multiple pathways, rather than obesity causing changes in the gut microbiome. Obesity mechanisms induced by the gut microbiota included energy absorption, fat storage, and chronic inflammation (Liu et al., 2021).

There is a close physiological connection between the mouth and the gut. Therefore, the association between oral microbiome and obesity is also worth studying (Mervish et al., 2019). The oral microbial community is more resilient than the gut microbiota toward exposure to antibiotics, diurnal variation, and diet (Stahringer et al., 2012; Cameron et al., 2015; Zaura et al., 2015; Belstrøm et al., 2016). However, research focusing on oral microbiota and obesity was not as much as that in the gut microbiota. Currently, most studies focus on cross-sectional populations, mainly changes in the oral microbiota of obese children (Zeigler et al., 2012; Balakrishnan et al., 2021; Rizzardi et al., 2021) and adults (Sohail et al., 2019; Yang et al., 2019). These studies have demonstrated that inflammatory changes in the oral cavity of obese patients are related to the oral microbiome (Abu-Shawish et al., 2022). On the other hand, there is a lack of animal studies. Li et al. analyzed the oral microbiome status in leptin-deficient obese mice (Li et al., 2023), and Chaves et al. investigated the impact of the oral microbiota on the alveolar bone regarding obesity (Chaves et al., 2022). Some researchers suggested that salivary microbiota may contribute to systemic metabolic changes, with specific oral bacteria potentially promoting insulin resistance by increasing tumor necrosis factor (TNF)-α and lipopolysaccharide levels or decreasing adiponectin levels, thereby altering energy expenditure and further influencing obesity (Coker et al., 2022). Additionally, the oral microbiome may affect food intake and obesity by regulating taste perception and appetite control (Coker et al., 2022).

Recently, the concept of the oral-gut axis has received widespread attention. Oral and gut microbiota were not isolated from each other, although it is unclear in what amount and proportion of ingested oral bacteria can penetrate the gastrointestinal defense barrier (Zeigler et al., 2012; Coker et al., 2022). Despite differences in the composition of the oral and gut microbiota, the types of communities observed in the gut could predict the types of communities observed in the oral cavity and vice versa (Ding and Schloss, 2014). Arimatsu et al. hypothesized that it is possible that swallowed bacteria in oral microbiota could affect the composition of the gut microflora (Arimatsu et al., 2014). Chen et al. proposed Helicobacter pylori infection can disrupt the normal gastric environment and enhance microbial interactions between the oral cavity and the gut (Chen et al., 2022). Abdelbary et al. found ectopic gut colonization by oral bacteria is increased in patients with inflammatory bowel disease (Abdelbary et al., 2022). It is pointed out that the oral–gut axis plays a significant role in the development of digestive system diseases or conditions related to digestion and metabolism (Ma et al., 2023). Chen et al. demonstrated a strong correlation between the oral-gut axis and clinical parameters related to hypertension, revealing the importance of oral-gut transmission of *Veillonella* in hypertension (Chen et al., 2023). Li et al. found an imbalance of the oral-gut axis in diabetes patients, and they proposed that targeting the oral-gut axis became an effective strategy to prevent and treat coronary heart disease in diabetes (Li et al., 2024a). Niu et al. believed that the oral-gut axis metastasis of *P. gingivalis* is an important mechanism connecting periodontitis and insulin resistance (Niu et al., 2024). The oral-gut axis is closely associated with metabolism, it might also contribute to obesity.

Therefore, the current study hypothesized that, similar to gut microbiota, salivary microbiota may also be involved in the pathophysiological process of obesity. This study investigated the composition of salivary and gut microbiotas and functional metabolite analysis in high-fat-fed mice.

## Materials and methods

### Study design

**Figure 1:**
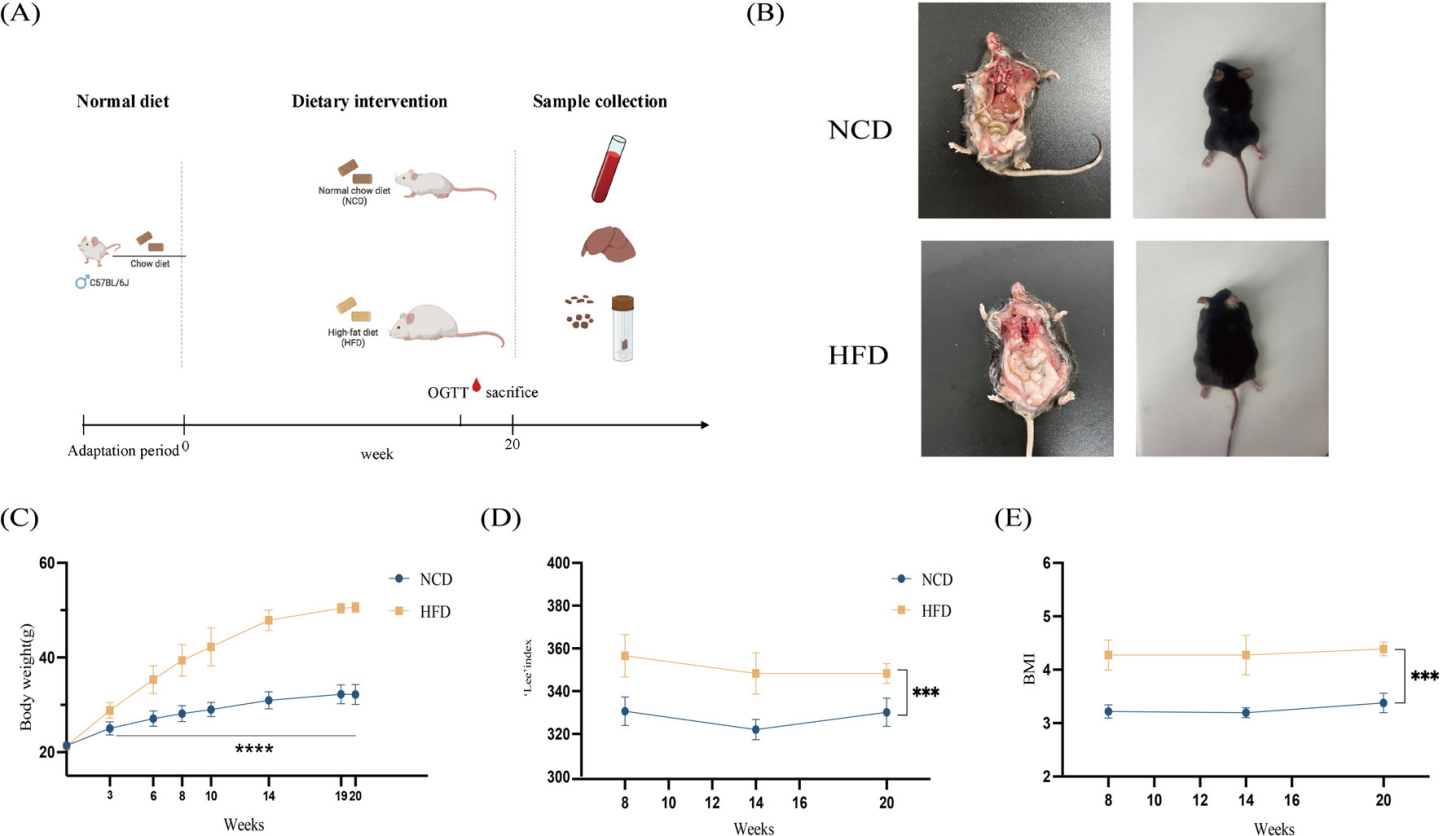
Schematic workflow diagram of the study. **(A)** Anthropometric measurements of mice in the HFD and NCD groups. **(B)** Representative dorsal and anatomical images of mice in the HFD and NCD groups at the 16th week of age; **(C)** Body weights of mice in the HFD and NCD groups; **(D)** Lee’s index in the HFD and NCD groups; **(E)** The body mass index (BMI) in the HFD and NCD groups. HFD, high-fat diet; NCD, normal-chow diet. ***p<0.001, ****p<0.0001.

The study design is illustrated in **Figure 1A**. This study utilized five-week-old specific pathogen-free C57BL/6 male mice (Jackson, USA). The sample size was set at 25/group regarding the previous research (Xiao et al., 2021), and the sample size of the HFD group was increased to 30 to ensure reliability. Mice were randomly allocated into two groups: the normal-chow diet group (NCD, n=25) and the high-fat diet group (HFD, n=30). After successfully establishing the obesity animal model, saliva, feces, blood, and tissues from both groups were collected. The study was approved by the Animal Protection and Use Committee of Peking University (DLASBD0276).

### Animal model

All the mice were housed with the condition of controlled temperature and humidity and a 12 h light/dark cycle, with ad libitum access to food and water. The animal house temperature and relative humidity were monitored daily. The two groups were housed separately, with no more than 5 mice per cage.

Body weight gain was assessed every two weeks. After 20 weeks of high-fat diet feeding, mice in the HFD group weighed greater than 20% of the NCD group, a criterion consistent with previous studies.

### Saliva and fecal sample collection

After the 20^th^ week, saliva and fecal samples were collected. Saliva samples were obtained by swabbing oral areas for 30s using sterile ultrafine cotton tips (Axygen, China) consistent with previous studies (Khurelchuluun et al., 2021). Samples were placed in 200µl of TE buffer individually and stored at -80°C until further analysis (Abusleme et al., 2017). Fecal samples were collected directly from stool expulsion stimulated by manual handling, of which 30 to 50mg were transferred into 1.5ml EP tubes and immediately frozen at -80°C until DNA extraction. All samples were collected consistently between 9 AM and 11 AM, avoiding the influence of concomitant circadian.

### Blood sample collection and analysis

Blood was collected by cardiac puncture with mice anesthetized by pentobarbital sodium, and serum was collected for additional analysis. Blood was transferred to EDTA microtainers (BD Biosciences) for counting and flow cytometry analysis using a fully automatic blood cell analyzer (BC-5000Vet Mindray, China). Plasma total cholesterol (TC), triglycerides (TG), high-density lipoprotein cholesterol (HDL-c), low-density lipoprotein cholesterol (LDL-c), and glucose (Glu) levels were measured using a Biochemical analyzer (model BS-430 apparatus, Mindra, China).

In the oral glucose tolerance test (OGTT), the mice were fasted for at least 16h followed by a glucose administration (2g/kg) by gavage. The blood glucose level was measured at 0, 15, 30, 60, and 120 min after the glucose load with one glucose meter (Sinocare GA-3, China).

### Tissue sample collection and pathological analysis

Tissue samples, including visceral fat pads, heart, liver, spleen, lungs, and kidneys were immediately removed after mice were sacrificed, weighed, and then fixed for 24 to 48h in 4% paraformaldehyde. The tissue specimens were embedded in paraffin wax, sliced to 4μm thickness, and placed on separate glass slides, dewaxed, rehydrated, and the hematoxylin-eosin (HE) staining was performed. The microscope (Nikon E100, Japan) was employed for capturing the final pictures (Niu et al., 2023).

### 16S rRNA gene sequencing

Total genomic DNA extraction was performed differently in saliva and fecal samples, determined by 0.8% agarose gel electrophoresis. DNA was quantified using a NanoDrop NC2000 spectrophotometer (Thermo Fisher Scientific, USA). Variable regions V3-V4 of the bacterial 16S rRNA gene, 338F(5’-barcode-ACTCCTACGGGAGGCAGCA-3’), 806R (5’-GGACTACHVGGGTWTCTAAT-3’) were amplified with degenerate PCR primers. The validated libraries were used for sequencing on an Illumina NovaSeq™ 6000 SP Reagent Kit (500 cycles) using paired-end 2 × 250 bp sequencing at Shanghai Personal Biotechnology Co., Ltd (Shanghai, China). Samples were sequenced in the same run to prevent batch effects.

### SCFA and MCFA analysis

Twenty mg of freeze-dried fecal samples were accurately weighed and placed in an EP tube, with 1mL of phosphoric acid (0.5% v/v) solution and a small steel ball added to the tube. The samples were ground uniformly, then vortexed for 10m and ultrasonicated for 5m. 100 μL of the mixture was centrifuged with a speed of 12,000 r/m for 10m at 4°C, and the supernatant was moved into a centrifugal tube afterward. 500μL of MTBE (containing internal standard) solution was added to the centrifugal tube and the mixture was vortexed for 3m followed by ultrasonicating for 5m. After that, the mixture was centrifuged at a speed of 12,000 r/min for 10 min at 4°C. The supernatant was collected and used for gas chromatography-tandem mass spectrometry (GC-MS/MS) analysis (Lee et al., 2014; Liu et al., 2017b; Li et al., 2020a, b).

Agilent 7890B gas chromatograph coupled to a 7000D mass spectrometer with a DB- FFAP column (30m length × 0.25mm i.d. × 0.25μm film thickness, J&W Scientific, USA) was employed for GC-MS/MS analysis of SCFAs and medium-chain fatty acids (MCFAs). Helium was used as carrier gas, at a flow rate of 1.2 mL/m. The injection was made in the split mode with a split ratio of 5:1 and the injection volume was 1μL. The oven temperature was held at 50°C for 1m, raised to 220°C at a rate of 18°C/min, and held for 5m. All samples were analyzed in multiple reaction monitoring mode. The injector inlet and transfer line temperature were 250°C and 230°C, respectively.

### Bioinformatics and statistical analysis

The microbiome biological information was analyzed using sequencing analysis software (QIIME2 2019.4, USA). The amplicon sequence variants (ASVs) characteristic sequences were compared with the reference sequences in the Greengenes2 database to obtain the taxonomic information corresponding to each ASV. The α diversity (within-sample diversity) was assessed using Shannon, Simpson, Pielou_e, Good’s coverage, observed species, Faith_pd, and Chao 1 indexes. Beta diversity (between-sample diversity) was measured with non-metric multidimensional scaling and principal coordinate analysis (PCoA). ANOSIM analysis with unifrac distance was applied to test for significant group cluster differences. Diagrams are visualized using R packages (version 4.3.0, Austria). The statistical significance of differentially abundant and biologically relevant taxonomical biomarkers between two distinct biological conditions was measured using a linear discriminant analysis (LDA) effect size (LEfSe). PICRUSt2 version 2.2.0 was used to predict the gut microbial metabolic functions based on the 16S sequences. Microbial functions were predicted using the Kyoto Encyclopedia of Genes and Genomes (KEGG) database.

Data are presented as the mean ± standard deviation unless otherwise indicated. One-way analysis of variance (ANOVA) with Bonferroni *post hoc* test (for parametric data sets) or Kruskal-Wallis test with Dunn’s multiple comparison test (for non-parametric data sets) were used to assign significance to the differences between groups. Two-way repeated measures ANOVA with a Bonferroni post-test was used when time was considered as a variable. Values of *p* < 0.05 were considered statistically significant. The statistical analyses were performed using GraphPad Prism (version 10.1.1, GraphPad Software), and SPSS (version 27.0, IBM).

## Results

### Physiological parameters of the HFD group

The body weight of mice in the HFD and NCD groups showed significant differences from the 3^rd^ week (*p*<0.001). From the eighth week, the body length of mice was measured regularly, and the body mass index (BMI) and Lee’s index were calculated. Compared with the NCD group, the BMI and Lee’s index of mice in the HFD group were significantly increased (*p*<0.001) (**Figure 1**).

**Figure 2:**
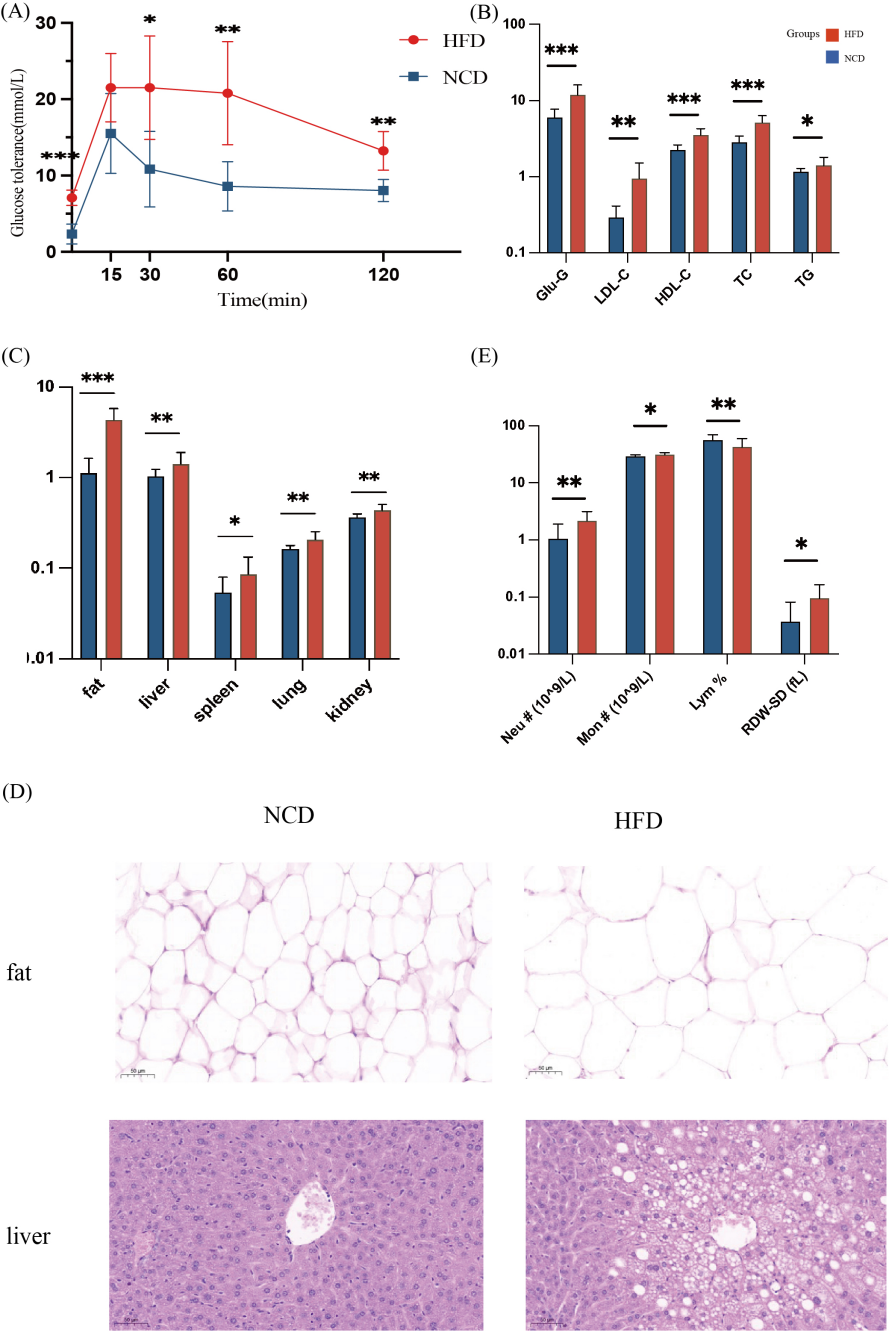
Physiological indicators of mice in the high-fat diet group (HFD) and the normal-chow diet group (NCD). **(A)** OGTT test of the HFD and the NCD groups: Mice were fasted for 16h with free drinking water. 25% glucose solution was used to intraperitoneally inject 2mg/kg glucose into the mice according to their body weight. The glucose level in the blood was measured using tail vein blood at 0m, 15m, 30m, 60m, and 120m, respectively; **(B)** Blood biochemical levels of the HFD and the NCD groups; **(C)** Inter-group comparison of fat-related organs and visceral adipose tissue; **(D)** H&E staining of liver and visceral fat sections (magnification 400 times); **(E)** Blood inflammation measurement, including the number of monocytes (Mon #), neutrophils (Neu #), the percentage of lymphocytes (Lym %), and the standard deviation of red blood cell distribution width (RDW-SD). *p<0.05, **p<0.01, ***p<0.001.

OGTT showed that the blood glucose concentrations of mice in the HFD group were statistically different from those in the NCD group when fasting 0m, 30m, 60m, and 120m later, with *p*<0.001, *p*=0.024, *p*=0.007, and *p*=0.004, respectively (**Figure 2A**). This study found that the blood glucose of the HFD group and the NCD group had significant differences (**Figure 2B**).

The study found significant inter-group blood lipid levels, with the serum TC, TG, HDL-c, and LDL-c in the HFD group significantly higher than those in the NCD group (**Figure 2B**). The weight of organs, including liver, spleen, lung, and kidney, and adipose tissue were significantly larger in the HFD group (**Figure 2C**). Changes in fat and liver cell morphology could be observed in the HFD and the NCD groups (**Figure 2D**). Histological examination of HE staining showed that compared with the NCD group, the size of adipocytes in the HFD group increased significantly, and the number of fat vacuoles in the same field of view was less (**Figure 2D**). The mice in the HFD group had significant hepatic steatosis, disordered arrangement of hepatic cords, enlarged hepatocytes, large round vacuoles in the cytoplasm, and the nuclei were squeezed to one side and deformed (**Figure 2D**).

The number of monocytes (Mon #), neutrophils (Neu #), and the standard deviation of red blood cell distribution width (RDW-SD) in the serum of mice in the HFD group were significantly higher than those in the NCD group. The percentage of lymphocytes (Lym %) was higher in the NCD group (**Figure 2E**).

High-fat diets induced weight gain, fat accumulation, and increased levels of serum glucose and lipid, and caused inflammatory conditions in mice.

### Diversity of saliva and gut microbiota

**Figure 3:**
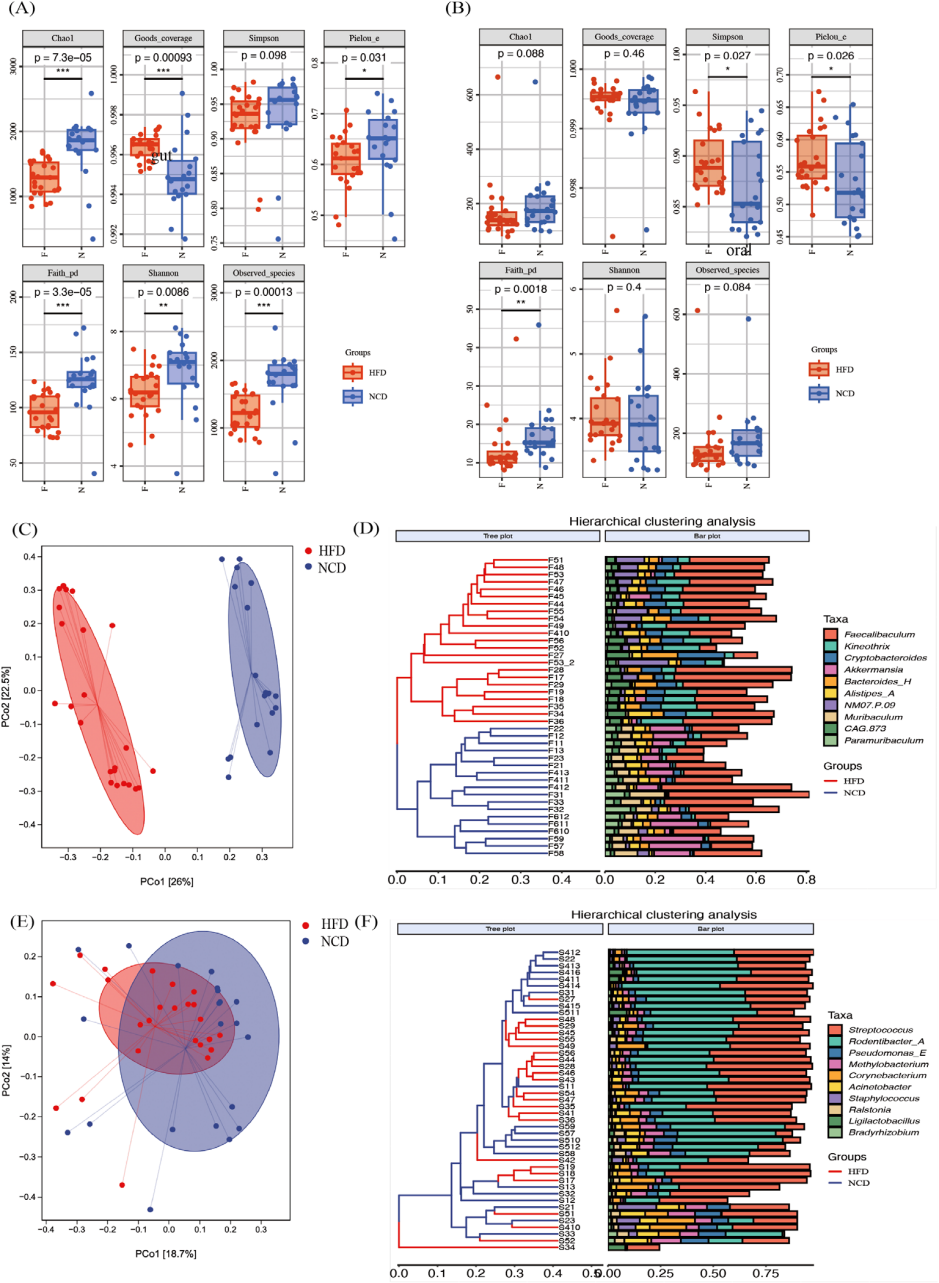
The α diversity and the β diversity indexes of the high-fat diet (HFD) and the normal-chow diet (NCD) groups. **(A)** The α diversity indexes of the gut microbiota; **(B)** The α diversity indexes of the saliva microbiota; **(C, D)** The principal coordinate analysis (PCoA) plot of gut microbiota based on Bray-Curtis’s distance and corresponding UPGMA hierarchical clustering plot; **(E, F)** The PCoA plot of salivary microbiota based on Bray-Curtis’s distance and corresponding UPGMA hierarchical clustering plot. *p<0.05, **p<0.01, ***p<0.001.

The α diversity indexes of the HFD and NCD groups are illustrated in **Figure 3**. Compared with the NCD group, the Chao1, Faith_pd, Shannon, Pielou_e, and Observed_species of the gut microbiota in the HFD group decreased significantly and the Good_coverage index increased significantly (**Figure 3A**). The Faith_pd index of the saliva microbiota in the HFD group decreased significantly, and the Simpson and Pielou_e indices increased (**Figure 3B**).

The alterations in the microbial community were investigated using PCoA based on Bray-Curtis’s distance (**Figures 3C–F**). The β diversity of the gut microbiota in the HFD and the NCD groups were significantly separated (**Figure 3C**, *p* = 0.001), with non-significant differences in variance within the groups (*p* = 0.586). The gut microbiota distribution of the HFD and the NCD groups were correctly clustered in the UPGMA cluster analysis diagram (**Figure 3D**). The β diversity of the salivary microbiota according to the Bray-Curtis distance was also statistically different between the two groups in PCoA, even though the separation was not clear (**Figure 3E**, *p* = 0.011). The UPGMA cluster analysis diagram in **Figure 3F** shows a mixing between the samples in the two groups.

Overall, the high-fat-induced obesity altered α and β diversity of the salivary and gut microbiota.

### Characteristic microbiota of the HFD group

**Figure 4:**
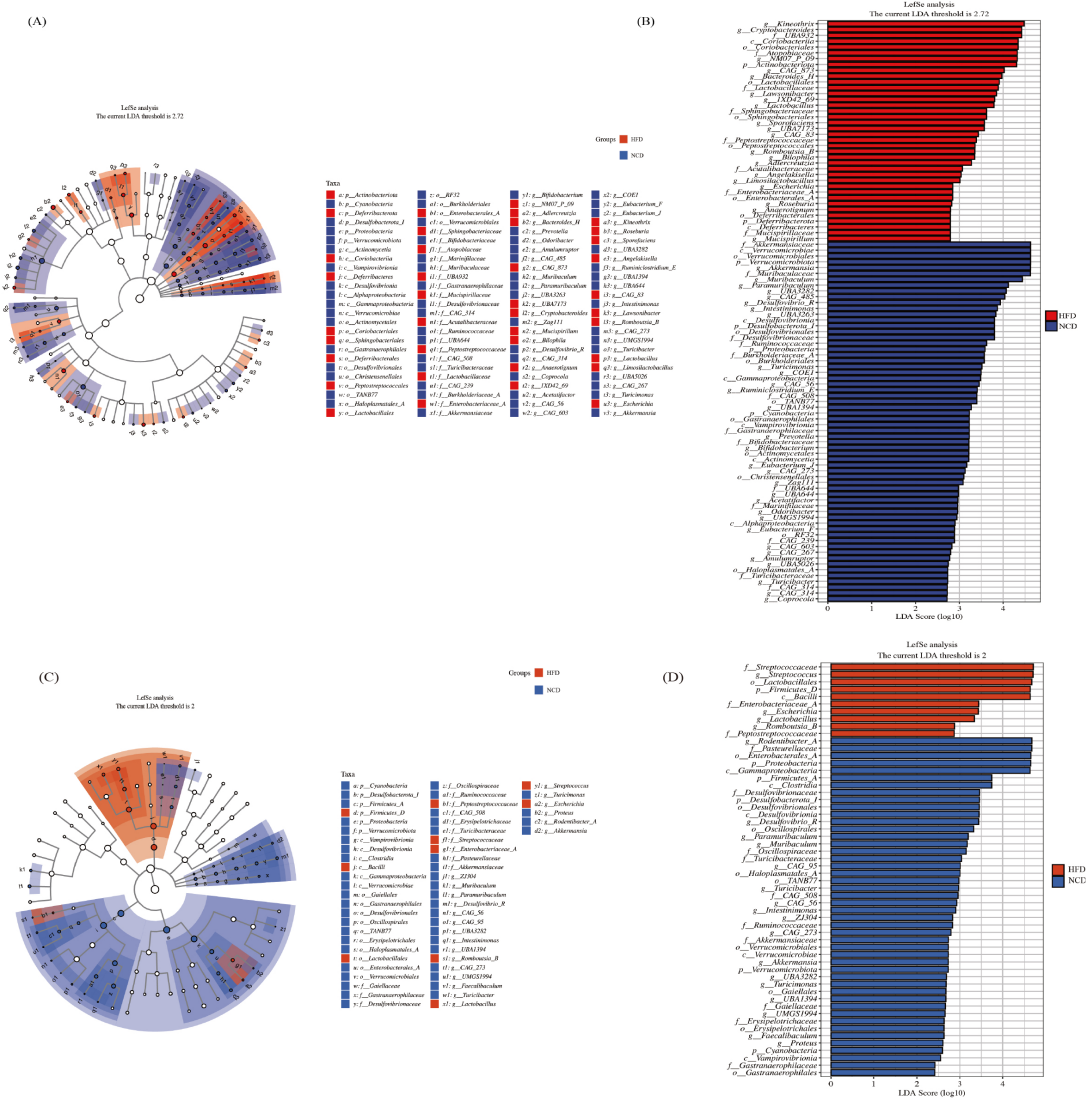
The LEfSe analysis of gut microbiota and salivary microbiota between the HFD and NCD groups. The cladogram **(A)** and rankogram **(B)** of the LEfSe analysis in gut microbiota; The cladogram **(C)** and rankogram **(D)** of the LEfSe analysis in salivary microbiota.

The LEfSe analysis was performed using the criteria of LDA score >2 and *p*<0.05 to select taxa that were significantly different between the HFD and NCD groups. There were more than 100 taxa detected in the gut microbiota, and the top 100 taxa are displayed in **Figures 4A, B**, among which, 38 taxa were enriched in the HFD group and 62 taxa were enriched in the NCD group. There were 56 taxa detected in the salivary microbiota (**Figures 4C, D**), with 10 taxa enriched in the HFD group and 46 taxa enriched in the NCD group.

**Figure 5:**
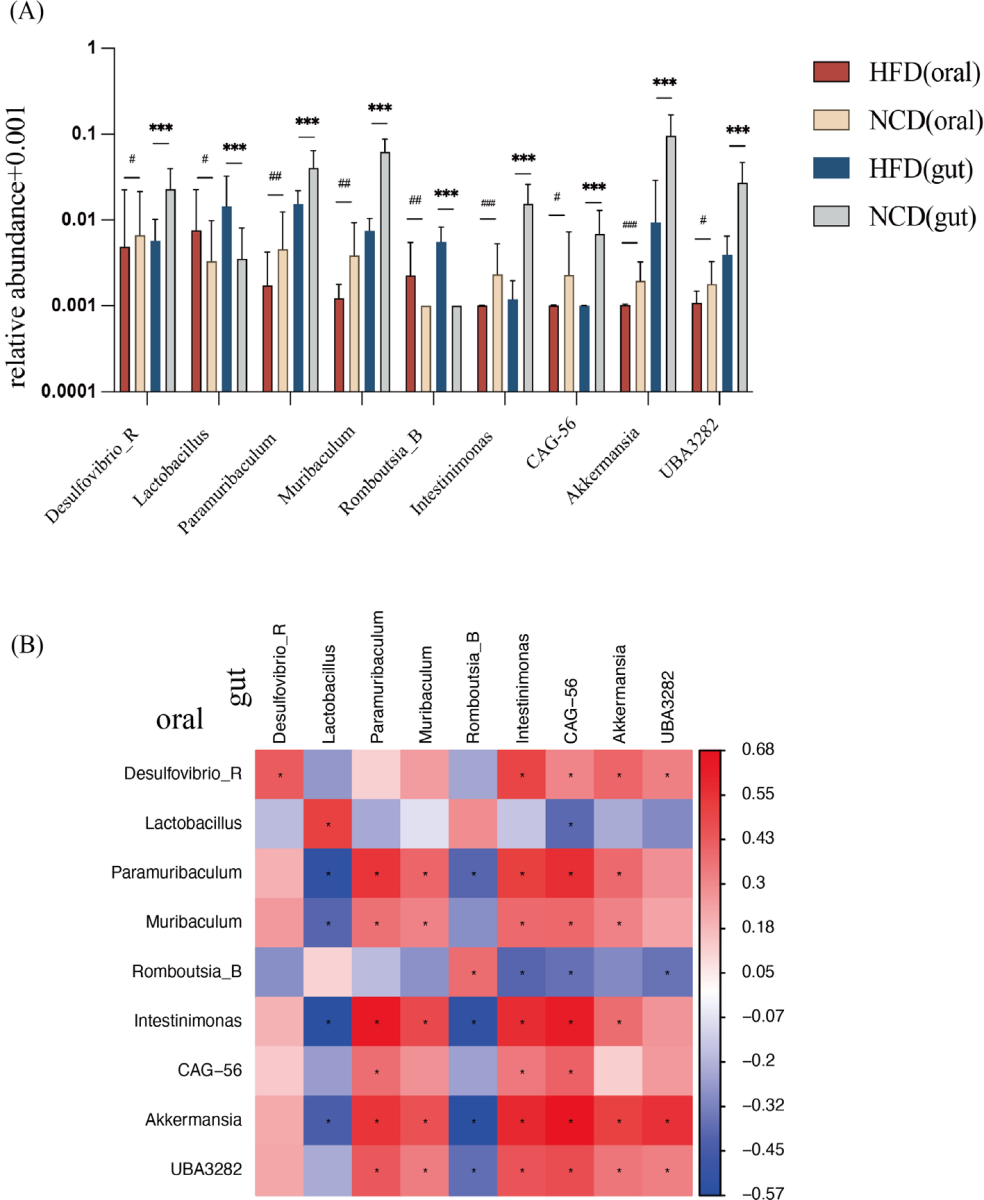
Relative abundance of nine genera with consistent significant differences in salivary and gut microbiota and correlation analysis. **(A)** Relative abundance of the nine genera with consistent significant differences. **(B)** Correlation analysis between the nine bacterial genera in salivary and in gut microbiota. *Significant difference in gut microbiota; ^#^Significant difference in salivary microbiota. */^#^ *p*<0.05, **/^##^ *p*<0.01, ***/^###^ *p*<0.001.

Compared with the NCD group, 9 genera in the HFD group had consistent alterations in both salivary and gut microbiota (**Figure 5A**). In the NCD group, the relative abundance of *Desulfovibrio_R*, *Muribaculum*, *Paramuribaculum*, *Intestinimonas*, *CAG-56*, *Akkermansia*, and *UBA3282* increased significantly; compared with the NCD group, the characteristic genera of the HFD microbiota were *Lactobacillus* and *Romboutsia_B*.

Correlation analysis between the salivary and gut microbiota was performed (**Figure 5B**). The abundance of each genus showed a positive correlation between salivary and gut microbiota. Meanwhile, *Akkermansia* in oral was negatively correlated with *Lactobacillus* in the gut, indicating a close relationship between oral and gut microbiota.

### Functional characteristics of the oral and gut microbiome

**Figure 6:**
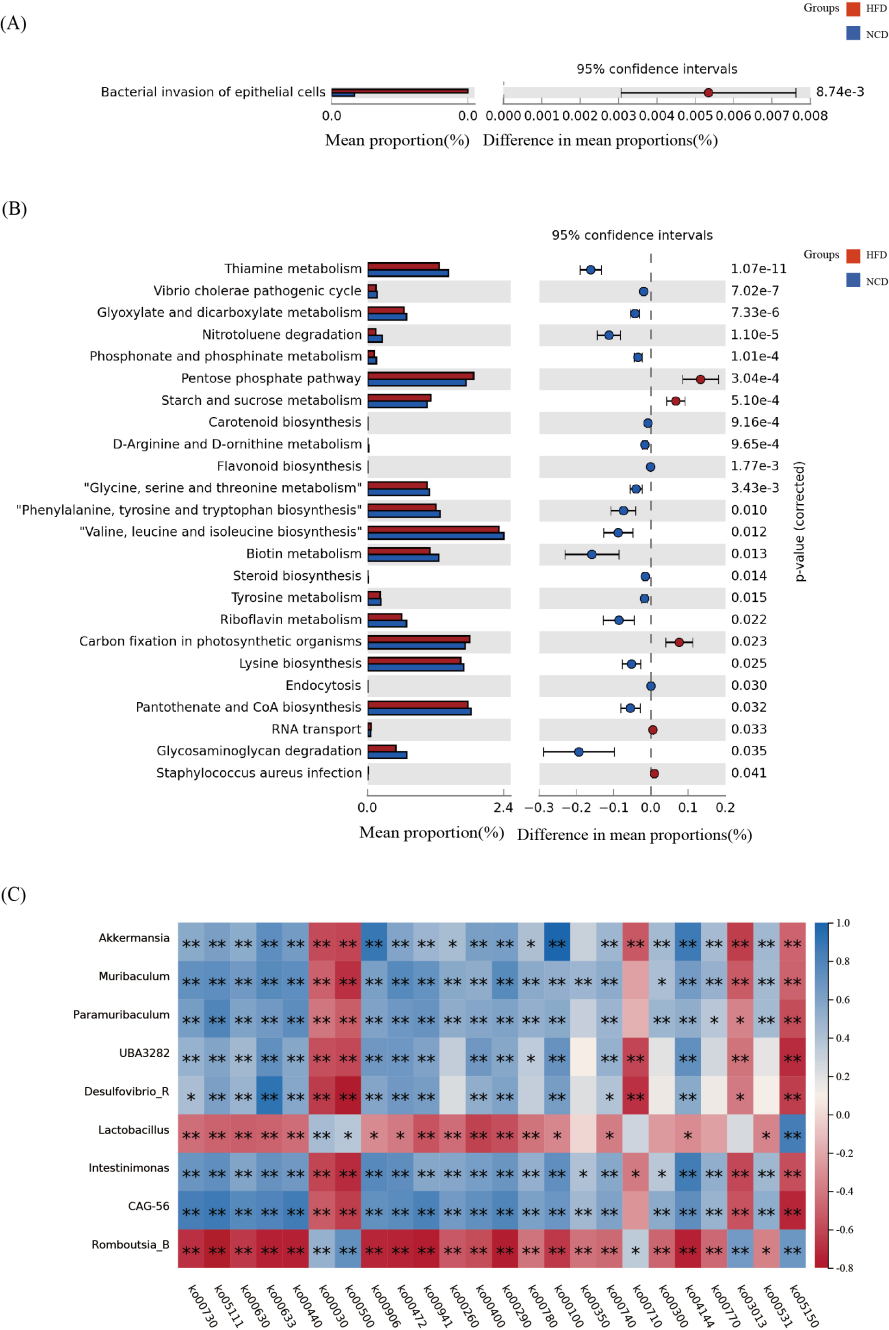
The functional metabolic pathways and correlation analysis. **(A)** The salivary microbial functions predicted using the KEGG database; **(B)** The gut microbial functions predicted using the KEGG database; **(C)** Correlation analysis between the functional metabolic pathways and the nine bacterial genera that were significantly different in the gut microbiota. *p<0.05, **p<0.01.

Microbial functions were predicted using the KEGG database to explore all potential pathways of the dysbiotic genera. This study identified one pathway with *p*<0.05 in the salivary microbiota, bacterial invasion of epithelial cells (ko05100), as shown in **Figure 6A**.There were 24 pathways identified in the gut microbiota (**Figure 6B**), including pentose phosphate pathway (ko00030), starch and sucrose metabolism (ko00500), glyoxylate and dicarboxylate metabolism (ko00630), glycosaminoglycan degradation (ko00531), steroid biosynthesis (ko00100), and thiamine metabolism (ko00730).

Furthermore, to investigate possible pathways of the oral-gut axis in inducing obesity, a correlation analysis was conducted between the nine genera that were consistently altered in the salivary and gut microbiota and the functional metabolic pathways (**Figure 6C**). It was found that *Lactobacillus* and *Romboutsia_B* were positively correlated with pentose phosphate pathway and starch and sucrose metabolism, while *Akkermansia*, *Muribaculum*, and other seven bacterial genera were negatively correlated. *Lactobacillus* and *Romboutsia_B* were negatively correlated with glycosaminoglycan degradation, steroid biosynthesis, and thiamine metabolism, while *Akkermansia*, *Muribaculum*, and others were positively correlated with glycosaminoglycan degradation, steroid biosynthesi, and thiamine metabolism.

A correlation analysis was performed between the bacterial invasion epithelial cells (ko05100) pathway and the 9 bacterial genera with significant differences in the saliva microbiota. It was found that *Muribaculum* (*p*=0.029, r^2^=-0.327), *Intestinimonas* (*p*=0.039, r^2^=-0.309), *Akkermansia* (*p*=0.001, r^2^=-0.498), and *UBA3282* (*p*=0.043, r^2^=-0.303) were significantly negatively correlated, and *Romboutsia_B* was significantly positively correlated (*p*=0.002, r^2^ = 0.444).

### SCFA and MCFA abundance in the HFD group

**Figure 7:**
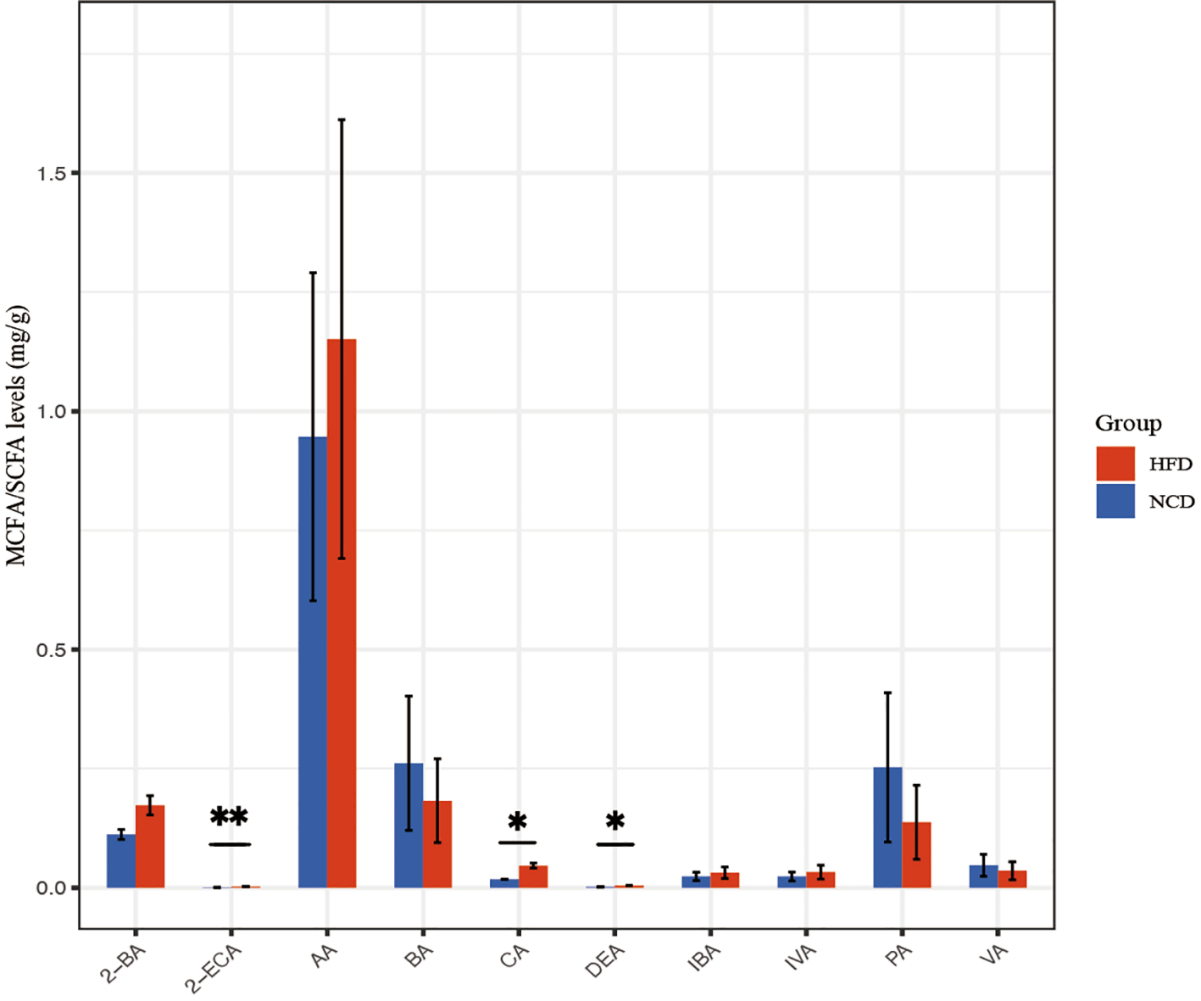
SCFA and MCFA abundance in the HFD and NCD groups. DEA, Decanoic acid; 2-ECA, 2-Ethylcaproic acid; 2-BA, 2-Methylbutyric acid; IVA, isovaleric acid; IBA, isobutyric acid; CA, caproic acid; BA, butyric acid; AA, acetic acid; PA, propionic acid; VA, valeric acid. *p<0.05, **p<0.01.

The abundances of SCFA and MCFA in fecal pellets were detected using non-targeted metabolomics (**Figure 7**). Metabolites with fold change ≥ 2 and *p*≤ 0.05 were selected. 2-ethyl caproic acid, caproic acid, and decanoic acid were significantly upregulated in the HFD group. The metabolites were annotated and displayed using the KEGG database. The fatty acid biosynthesis pathway and metabolic pathways were significantly upregulated in the HFD group related to decanoic acid.

### Correlation between salivary and gut microbiota and physiological parameters

**Figure 8:**
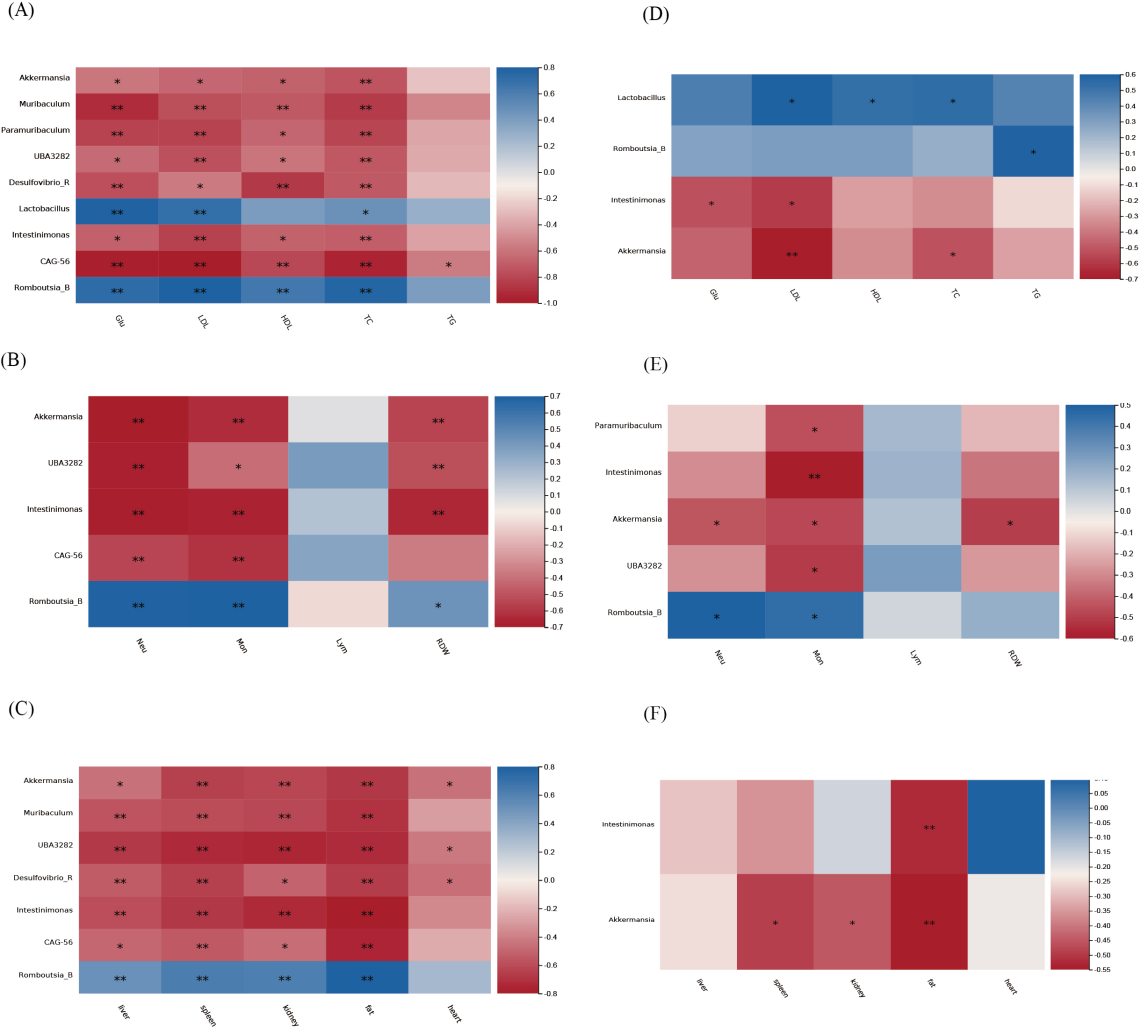
Correlation analysis between physiological parameters and the nine genera in the HFD group with consistent alterations in both salivary and gut microbiota. **(A-C)** Gut microbiota; **(D-F)** Salivary microbiota.

The correlation analysis between physiological parameters and the nine genera in the HFD group with consistent alterations in both salivary and gut microbiota is shown in **Figure 8**. In the gut microbiota (**Figures 8A–C**), *Akkermansia*, *UBA3282*, and *Intestinimona* were negatively correlated with organs’ weights, Glu, TC, HDL, LDL, Mon #, Neu #, and RDW-SD. *Akkermansia*, *UBA3282*, and *Intestinimona* were negatively correlated with Mon # and Neu #. *Muribaculum* and *CAG-56* were strongly negatively correlated with glucose and lipid metabolism parameters, such as Glu and LDL. *Romboutsia_B* was statistically positively correlated with organs’ weights, Glu, TC, HDL, LDL, Mon #, Neu #, and RDW-SD and *Lactobacillus* was positively correlated with TC, LDL, and Glu. In the salivary microbiota (**Figures 8D, E**), *Akkermansia* and *Intestinimona* were negatively correlated with TC, LDL, Mon #, and organs’ weights. *Romboutsia_*B was positively correlated with TG, Mon #, and Neu #, whereas *Lactobacillus* was positively correlated with HDL, LDL, and TC.

## Discussion

Obesity is typically a reflection of an imbalance between food intake and energy expenditure and is influenced by genetic and environmental factors (Kahn et al., 2006). High-fat diets induce obesity through gut microbiota in a unidirectional manner (Hildebrandt et al., 2009; Murphy et al., 2010; Lee et al., 2020). There might be an association between salivary and gut microbiome through the oral-gut axis. However, whether similar changes occur in the salivary microbiota in obesity remains insufficiently studied. This study was designed to investigate the similarities and differences in the effects of a high-fat diet on salivary and gut microbiota through mouse experiments, exploring the hypothesis that oral microbial mechanisms may contribute to obesity. Using 16S rRNA sequencing technology, this study analyzed changes in salivary and gut microbiota, and GC-MS was employed to detect 17 SCFAs and MCFAs quantitatively. Through these methods, the study identified diet-induced alterations in the salivary and gut microbiota of mice, including characteristic genera such as *Akkermansia* and *Muribaculum.* Furthermore, the study explored potential pathways through which these distinct genera might contribute to obesity. To our knowledge, this is the first study to integrate multiple biochemical indicators to analyze differences in both oral and gut microbiota and their association with obesity in mice.

### The impact of the high-fat diet on salivary and gut microbiota

Given the complex interactions established between the salivary and gut microbiota and their host, the salivary and gut microbiomes have emerged as core players in human health and disease (Kunath et al., 2024). Understanding the oral-gut microbiome axis and the bidirectional interaction between these microbial communities is crucial for elucidating their impact on host physiology and pathology (Kunath et al., 2024).

This study found that the high-fat diet significantly altered the α diversity in both salivary and gut microbiota, as evidenced by decreased Faith_ PD, indicating reduced evolutionary diversity in microbiota. However, reductions in indices such as Chao1 and Shannon, observed in the gut microbiota of the HFD group, were not seen in the salivary microbiota. The Pielou_e decreased in the gut microbiota but increased in the salivary microbiota, suggesting distinct changes in species evenness between the two microbiomes. Similarly, β diversity underwent significant changes in both salivary and gut microbiota in the HFD group, with a more pronounced separation between HFD and NCD groups in the gut microbiota, as confirmed by clustering analysis. Conversely, the separation trend was weaker in the salivary microbiota. Overall, while a high-fat diet affected the diversity of salivary and gut microbiota similarly, the effects were stronger on the gut microbiota, possibly due to its lower stability compared to the salivary microbiota (Raju et al., 2019). Shoer et al. also found that dietary interventions caused greater compositional changes in the gut microbiome than in the oral microbiome (Shoer et al., 2023). Although no previous studies have examined the effects of a high-fat diet on the diversity of oral microbiota in mice, findings in obese human populations remain contradictory, with α and β diversity reported to increase, decrease, or remain unchanged (Craig et al., 2018; Mameli et al., 2019; Rizzardi et al., 2021; Coker et al., 2022; Ma et al., 2023). However, consistent with this study, prior research has shown that a high-fat diet reduces Chao1, Shannon, and Simpson indices in mouse gut microbiota (Anhê et al., 2019; Wei et al., 2024).

Despite the anatomical connection between the oral and gut regions, their microbial communities are distinct and share limited taxa (Kunath et al., 2024). This study revealed that a high-fat diet altered the abundance of certain genera in both salivary and gut microbiota. Seven genera, including *Akkermansia*, *Muribaculum*, and *Intestinimonas*, showed decreased abundance in both salivary and gut microbiota under HFD, while *Lactobacillus* and *Romboutsia_B* increased in abundance in both microbiota communities. No genera exhibited divergent abundance trends between the salivary and gut microbiota.

Additionally, the abundance of *Akkermansia* and *Intestinimonas* in both salivary and gut microbiota showed significant negative correlations with inflammation and metabolic parameters in mice, while HFD-characteristic genera such as *Lactobacillus* were positively correlated with blood lipid levels, and *Romboutsia_B* was positively correlated with inflammatory markers. The similar changes in abundance and associations with physiological indicators suggest a potential bidirectional relationship between the oral and gut microbiota. Kitamoto et al. showed that dietary changes increased the relative abundance of oral bacteria in fecal samples (Kitamoto et al., 2020a), and Bao et al. reported that periodontitis could induce alterations in gut microbiota through oral microbial translocation (Bao et al., 2022). Under physiological conditions, defensive mechanisms such as the oral-gut barrier, gastric acid, bile, and colonization resistance of the gut microbiota limit the spread of oral bacteria to the gut. However, under certain pathological conditions, oral bacterial translocation increases, leading to gut microbiota dysbiosis and disruption of gut homeostasis (Assimakopoulos et al., 2018; Al-Qadami et al., 2022; Kunath et al., 2024).

### Mechanisms of the oral-gut axis in inducing obesity

This study found significant correlations between oral and gut microbiome. Oral bacteria can disseminate to the gastrointestinal tract via hematogenous or intestinal routes, eliciting abnormal immune responses and resulting in intestinal inflammation (Saygun et al., 2011; Kitamoto et al., 2020b). The oral microbiome may affect the bacteria that colonize the gut microbiome (Nakajima et al., 2015). Currently, extensive research has revealed the role of the oral-gut axis in the mechanism of various diseases, including diabetes, rheumatoid arthritis, non-alcoholic fatty liver disease, inflammatory bowel disease, pancreatic cancer, and colorectal cancer (Tan et al., 2023).

The gut microbiota plays a critical role in mediating the influence of oral microbiota on diabetes (Shen et al., 2022). Numerous studies on both adults and mice have demonstrated a strong association between the gut microbiota and obesity (Ma et al., 2023). Consequently, this study focused on salivary microbiota that exhibits synergistic changes with the gut microbiota of obesity.

Our study found that the abundance of *Akkermansia* in both salivary and gut was significantly reduced in the HFD group. Additionally, this reduction correlated negatively with obesity-related parameters, such as adipose tissue weight, TC, and LDL. While causal evidence linking *Akkermansia* to obesity remains limited (Li et al., 2024b), previous animal and human studies have associated obesity with reduced gut *Akkermansia* levels (Cani et al., 2022), both in clinical research and animal experiments. Dao et al. found that higher *Akkermansia muciniphila* abundance is associated with a healthier metabolic status in obese humans (Dao et al., 2016). *Akkermansia* was associated with normal weight (De-La-Cuesta-Zuluaga et al., 2018). Studies have identified a loss in abundance of *Akkermansia muciniphila* in patients with obesity (Everard et al., 2013). Moreover, a decrease in *Akkermansia* was observed in the salivary microbiota of leptin-deficient ob/ob mice (Li et al., 2023). Dong et al. demonstrated that administering *P.gingivalis* resulted in metabolic disturbances similar to those in HFD mice, including adipocyte hypertrophy, macrophage infiltration, gut barrier defects, and insulin resistance (Dong et al., 2022). Cani et al. were the first to show that daily administration of live *Akkermansia muciniphila* in mice reversed HFD-induced metabolic disorders through mechanisms such as the production of extracellular vesicles that alleviate gut inflammation, stimulation of mucus secretion by Amuc_1100 protein, and regulation of glucose homeostasis by promoting glucagon-like peptide-1 and 2 secretion via outer membrane protein P9 (Cani et al., 2022). Previous studies suggested the following potential mechanisms by which *Akkermansia* may contribute to obesity:

Firstly, *Akkermansia* might be associated with obesity through the pentose phosphate pathway. The functional and correlational analyses in the current study revealed a negative correlation between gut *Akkermansia* abundance and the pentose phosphate pathway. This pathway, a key glucose catabolic process, supports *de novo* fatty acid synthesis in adipose tissue by generating nicotinamide adenine dinucleotide phosphate (Zhang et al., 2021), which reduces metabolic reactions such as fatty acid and cholesterol biosynthesis (Tian et al., 2020). Thus, a decrease in *Akkermansia* abundance may enhance the pentose phosphate pathway, leading to increased synthesis of fatty acids and sterols.

Secondly, *Akkermansia* might promote inflammation in the gut and bloodstream to contribute to obesity. This study observed a positive correlation between gut *Akkermansia* abundance and the thiamine metabolism pathway, consistent with findings by Hui et al (Hui et al., 2024). They demonstrated that modulating *Akkermansia* impacts thiamine metabolism and inhibits macrophage activation, thereby regulating NF-κB/Nrf2/COX-2-mediated inflammation and oxidative stress in inflammatory bowel disease. In the HFD group, reduced *Akkermansia* levels may lower thiamine metabolism activity, promoting gut inflammation. Similarly, decreased oral *Akkermansia* levels may activate bacterial invasion of epithelial cells, a key event in inflammation initiation. Our data showed increased monocyte (Mon #) and neutrophil (Neu #) counts and RDW-SD, alongside reduced lymphocyte percentage (Lym %) in the HFD group. Several studies have linked these hematological parameters to obesity (Trottier et al., 2012; Cui et al., 2020; Cohen et al., 2021; Christakoudi et al., 2023; Klisic et al., 2023). Li et al. concluded through Mendelian randomization that neutrophil count, platelet count, reticulocyte count, and white blood cell count have positive causal relationships with obesity (Li et al., 2024b).

Other genus, including *Muribaculum* and *Paramuribaculum*, might be associated with activating fatty acid synthesis pathways, leading to obesity. This study observed significant negative correlations between the abundance of *Muribaculum* and *Paramuribaculum* and obesity-related parameters, such as adipose tissue weight. These genera, both members of the family Muribaculaceae within the order Bacteroidales, share similarities with *Akkermansia* in degrading mucus (Glover et al., 2022; Berlin et al., 2023). Studies by Song et al. (Song et al., 2021) and Do et al. (Do et al., 2018) have shown strong associations between *Muribaculum* and host obesity and metabolic disorders. Xu et al. suggested that a reduction in *Muribaculum* due to an HFD decreases intestinal hyodeoxycholic acid (HDCA) levels, increasing lipid absorption via the gut FXR-FGF19 axis (Xu et al., 2023). Additionally, *Muribaculum* produces SCFAs, which our study found to correlate significantly with increased decanoic acid levels in the HFD group. KEGG annotation and enrichment analyses of decanoic acid revealed activation of fatty acid biosynthesis and metabolic pathways in the HFD group. These findings align with research indicating that dysregulated fatty acid biosynthesis contributes to obesity and non-alcoholic fatty liver disease (Günenc et al., 2022). Activation of fatty acid biosynthesis pathways may, therefore, mediate the role of *Muribaculum* in obesity.

The current study has several strengths. Firstly, unlike previous studies of obese mice that primarily focused on gut microbiota, the present study is, to the best of our knowledge, the first to simultaneously analyze the differences between oral and gut microbiota, and their mutual regulation in high-fat diet-induced obese mice. Specific microbiota were identified, such as *Akkermasia*, existed in both the oral and gut, and how its distribution was altered. Secondly, this study integrated data on glucose metabolism, lipid metabolism, and inflammatory indexes with oral and gut microbiota profiles in mice. The study found significant associations suggesting that oral microbiota may play a role in obesity, similar to gut microbiota. However, there are also some limitations. This study utilized routine blood parameters such as white blood cell count as preliminary exploration, whereas it would be more reasonable to measure interleukin-6 (IL-6), IL-1, and other markers of blood inflammation using western blot in mice. This study lacks a causal relationship validation. Besides, there are inherent differences between mouse’s and human’s microbiota. The current study was based on a mouse model, and the identified characteristic bacterial genera may differ from those found in humans. We will conduct further validation using methods such as microbiota transplantation in the future.

In conclusion, this study suggested that while a high-fat diet affected the diversity of salivary and gut microbiota similarly, the effects were stronger on the gut microbiota. There were 9 genera in the HFD group with consistent alterations in both salivary and gut microbiota; however, some genera showed changes exclusively in either the oral or gut microbiome. Oral and gut microbiota, particularly *Akkermansia*, *Muribaculum*, and *Paramuribaculum*, may influence obesity through mechanisms involving metabolic pathways, inflammation, and fatty acid biosynthesis. Further research is warranted to validate these findings and explore therapeutic interventions targeting the oral-gut microbiota axis.

## Data Availability

The data presented in the study are deposited in the Sequence Read Archive (https://www.ncbi.nlm.nih.gov/) with the accession number PRJNA1216042.
